# Current practice of glucocorticoid replacement therapy and patient-perceived health outcomes in adrenal insufficiency - a worldwide patient survey

**DOI:** 10.1186/1472-6823-12-8

**Published:** 2012-06-13

**Authors:** M Forss, G Batcheller, S Skrtic, G Johannsson

**Affiliations:** 1 DuoCort Pharma, Medicinaregatan 8A, 413 46, Gothenburg, Sweden; 2 Department of Clinical Pharmacology, Sahlgrenska Academy, University of Gothenburg, Gothenburg, Sweden; 3 Department of Endocrinology, Sahlgrenska Academy, University of Gothenburg, Gothenburg, Sweden

## Abstract

**Background:**

The aim was to survey current practice in glucocorticoid replacement therapy and self-perceived health outcomes in patients with adrenal insufficiency.

**Methods:**

Participants were recruited via patient organizations to respond anonymously to a web-based survey developed by clinical experts. Unique entries were set up for each patient organization enabling geographical localization of the entries.

**Results:**

1245 participants responded (primary adrenal insufficiency: 84%; secondary adrenal insufficiency: 11%; unsure: 5%). Therapies included hydrocortisone (75%), prednisone/prednisolone (11%), cortisone acetate (6%) and dexamethasone (4%). Dosing regimens were once daily (10%), twice daily (42%), thrice daily (32%) or other (17%). Compromised subjective health necessitating changes to physical activity or social-, work- or family life was reported by 64% of the participants. 40% of the participants reported absence from work/school in the last 3 months. Irrespective of diagnosis, 76% were concerned about long-term side-effects of therapy, mainly osteoporosis (78%), obesity (64%) and cardiovascular morbidity (46%). 38% of the participants had been hospitalized in the last year.

**Conclusions:**

Glucocorticoid replacement therapy among the respondents consisted primarily of hydrocortisone administered twice or thrice daily. A majority reported impact of their disease or treatment on subjective health requiring alterations in e.g. physical activity or family life. Three quarters reported concerns about long-term side-effects of the treatment. These data demonstrate - from the patients' perspective - a need for improvement in the management of adrenal insufficiency.

## Background

Recent data indicate that replacement therapy with glucocorticoids in adrenal insufficiency (AI) is associated with poor long-term outcome. In the two largest registry-based studies on mortality in patients with Addison's disease, the relative risk of death was more than 2-fold compared to the background population despite treatment [1,2]. Patients with hypopituitarism and secondary AI also have increased relative risk of death [3]. Based on the increase in cardiovascular risk factors [4,5] and reduced bone mineral density (mainly in female AI patients) [6-10], overly high glucocorticoid exposure is likely to occur at least during parts of the day/night. This is supported by data showing an inverse relationship between dose exposure and bone mineral density [7]. Due to the short half-life of hydrocortisone, each oral dose is followed by a rapid increase and often an overly high peak in serum cortisol concentration followed by a rapid decline. Multiple doses of immediate-release hydrocortisone tablets are needed in order to cover the active part of the day but result in a peak after each dose and a trough between doses. It has been estimated that AI patients are on average over-substituted by 6-7 mg hydrocortisone/day based on an ideal body mass area-dose ratio of 11 mg/m^2^[11]. Besides symptoms indicating over-exposure to glucocorticoids, e.g. a tendency to gain weight, symptoms indicating underexposure, e.g. salt craving, were also commonly reported in a previous survey [12].

The subjective health-related quality of life (QoL) of AI patients is impaired on a group level [13-16] with health scores inferior to those of the background population. General health and vitality have been found to be consistently impaired [13], with fatigue being the most characteristic subjective health feature of AI [12,13]. Working ability is reduced to various degrees in different countries [11-14]. Attempts to re-establish the normal cortisol exposure-time profile have in an open study demonstrated improvement in well-being [17], and improved QoL was observed in another study when changing from a twice daily (BID) to a thrice daily (TID) regimen [18]. These data suggest that the peaks and troughs between doses may to some extent explain the poor self-perceived well-being reported by patients.

The aim of this patient survey was to investigate current practice in glucocorticoid replacement therapy (therapy and dosage regimen) in patients with primary or secondary AI in different countries and the participants’ self-perceived health status and outcomes by type of disease and therapy.

## Methods

This was an open cross-sectional survey. Participants were recruited via patient organizations (e-mail contact lists and newsletters) to respond anonymously to the web-based survey. The following patient organizations actively approached their members to participate in the survey and had links to the survey on their home page: National Adrenal Diseases Foundation (NADF) in the US, Australian Addison’s Disease Foundation, Addison’s Disease Self Help Group (ADSHG) in the UK and Cushing’s Support and Research Foundation (CSRF) in the US. Some additional patient organizations also had information about and/or links to the survey on their respective websites: CARES foundation in the US, Association Surrénales in France, two Swedish associations (The Swedish Addison Association and Hypofysis), two Danish associations (Addisonforeningen in Denmark and Danish Morbus Addison Site), the Dutch Addison & Cushing Society (NVACP) and Associazioni Italiana Pazienti Addison in Italy. A link was also set up on the sponsor’s website for other patients wanting to complete the survey.

The questionnaire covered a range of questions (39 questions in total), including patient demographics (country, type of disease, etc.), medication and satisfaction with the current medication. It also covered impact of the disease on self-perceived subjective health, the prevalence and impact of fatigue, views on long-term side effects related to treatment, hospitalizations and absenteeism from work or school. The questionnaire consisted of a mix of single select, multiple select and open questions. A pilot survey was conducted with four members from different patient organizations to ensure the validity of the questions in the questionnaire. Revisions were then made to the questionnaire before the survey was conducted. In order to ensure the privacy of the participants’ contact details, it was agreed that each patient organization should send out alerts via e-mail and newsletters to inform its members about the survey and its objectives. Although no research ethics committee approval is needed for the conduct of this kind of patient survey [19], a research application had to be completed for the UK patient organization ADSHG, in order to be able to recruit volunteers from their membership registry. No remuneration was given to the participants. No data on age and gender were collected in order to further protect the individual identity of the participants.

Unique links for each patient organization were set up in the web survey tool Easyresearch (by QuestBack, Oslo, Norway), the technical platform used for administration and analysis of the survey. The unique entries enabled geographical localization of the entries. The intention was to have the survey open for approximately 6 weeks, but the survey opening time was extended as a result of the high degree of interest from individual patients and additional patient organizations. The survey was open from September 12^th^ to December 19^th^ 2008.

The data were analyzed descriptively (frequency analysis) by disease type (primary or secondary AI), treatment and dosing regimen. The country-specific analyses included only countries with more than 20 participants. The denominator for the calculation of frequency varied between the questions since not all participants answered all questions. The asking of some of the questions, such as on subjective general health, was dependent on previous answers (e.g. degree of impact on QoL was only asked if the participant had answered that the disease affected their QoL). No statistical tests were performed.

## Results

A total of 1281 persons visited the webpage of the survey whereof 1245 responded to at least the first question (“In which country do you live?”). The respondents were from (by number of participants): US (801), Australia (90), France (81), UK (80), Canada (37), Sweden (35), Denmark (19), the Netherlands (8), Germany (7), Belgium (6), New Zealand (5), Mexico (4), Ecuador (3), Ireland (3), Spain (3), Chile (2), Dominican Republic (2), India (2), Norway (2), Philippines (2), Poland (2), South Africa (2), Switzerland (2), Argentina (1), Dubai (1), Greece (1), Hungary (1), Israel (1), Italy (1), Jamaica (1), Martinique (1), Portugal (1), Serbia (1) and Uruguay (1).

When asked “What type of cortisol deficiency are you suffering from?”, 939 participants (84%) defined their AI as primary (Addison’s disease, congenital adrenal hyperplasia, adrenal disease causing dysfunction of adrenal glands or removed glands) and 125 (11%) as secondary (pituitary or hypothalamic disease) while 51 (5%) were unsure.

### Glucocorticoid treatment regimen

Overall, hydrocortisone was used by 75% of the participants, prednisone/prednisolone by 11%, cortisone acetate by 6% and dexamethasone by 4% of the participants, Table 1. A high proportion of participants (40%) in Australia reported using cortisone acetate. Among the countries with at least 20 respondents, the use of prednisone/prednisolone was most common in Canada (27%), the US (14%) and Australia (11%) and dexamethasone was most commonly used in Australia (5%) and the US (4%). The distribution of therapies was similar between patients with primary and secondary AI. The majority of the patients were on BID (42%), or TID (32%), while 10% were on an OD regimen, Table 1. Of the patients on a BID regimen, 58% took their medication in the morning and afternoon and 42% in the morning and evening. Patients with secondary AI took their BID dosing in the morning and afternoon (39%) rather than in the morning and evening (9%). 53% of the patients on prednisone/prednisolone were on BID and 5% on TID. For treatment regimen by therapy, please see Table 2.

**Table 1:** Therapy and dosing regimen, by type of adrenal insufficiency, reported in an international patient survey

		**Primary AI**	**Secondary AI**	**All**
		**n (%)**	**n (%)**	**n (%)**
Therapy	Hydrocortisone	697 (75%)	98 (80%)	833 (75%)
	Prednisone, prednisolone	100 (11%)	12 (10%)	124 (11%)
	Cortisone acetate	57 (6%)	4 (3%)	63 (6%)
	Dexamethasone	35 (4%)	4 (3%)	39 (4%)
	Other	38 (4%)	4 (3%)	50 (5%)
	N	N = 927	N = 122	N = 1109
Dosing regimen	Once daily	80 (9%)	14 (12%)	104 (10%)
	Twice daily	360 (42%)	58 (48%)	437 (42%)
	Thrice daily	291 (34%)	25 (21%)	328 (32%)
	Other	135 (16%)	23 (19%)	173 (17%)
	N	N = 866	N = 120	N = 1042

Note: The number of patients responding to the question about therapy was not the same as the number of patients reporting dosing regimen (different questions).

**Table 2:** Dosing regimen, by therapy, reported in an international patient survey

	**Hydrocortisone**	**Prednisone/prednisolone**	**Cortisone acetate**	All
	**n (%)**	**n (%)**	**n (%)**	**n (%)**
	**(N = 772)**	**(N = 116)**	**(N = 60)**	**(N = 1042)**
Once daily	47 (6%)	35 (30%)	4 (7%)	104 (10%)
Twice daily	304 (39%)	61 (53%)	44 (73%)	437 (42%)
Thrice daily	301 (39%)	6 (5%)	8 (13%)	328 (32%)
Other	120 (16%)	14 (12%)	4 (7%)	173 (17%)

Note: The number of patients recording dosing regimen was not the same as the number of patients reporting therapy (Table 1).

One quarter (23%) of the participants were dissatisfied or very dissatisfied with their current treatment, 18% were indifferent and 59% were satisfied or very satisfied. Patients with secondary AI reported less satisfaction with their current therapy than patients with primary AI. The ratings of satisfaction were similar among the different therapies.

Multiple daily dosing was reported as a problem by 38% of the participants whereof 15% were on OD, 35% on BID, 32% on TID and 17% on another regimen. Of those who did not find multiple daily dosing to be a problem 7% were on OD, 45% on BID, 32% on TID and 16% on another regimen. Among respondents answering that multiple daily dosing was a problem 94% reported one or more of the following: difficulties to remember/forgetting doses (particularly the midday and afternoon doses), difficulties in taking the medication at a specific time every day and/or difficulties to remember to bring the medication. Many reported that their days are planned according to their dose intake and for those who lead a busy life and are working, this was found to be challenging as some also reported that they do not want to be seen by their colleagues when taking their medication. Several reported that multiple daily dosing becomes very restrictive to an active life and that multiple daily dosing is a reminder several times per day that they have this disease. Many also reported health issues such as being fatigue and exhausted in the day, evening and the following day(s) if missing a dose. In addition, many reported that taking a missed dose too late disrupts their sleep and causes issues with insomnia. Some reported instability of physical and mental well-being with mood swings and ups and downs in energy levels. The patients who reported multiple daily dosing to be a problem also reported higher frequencies of impacted QoL, more fatigue and more activities altered due to their disease (data not shown).

Enduring efficacy over 24 hours was considered the most important feature of an optimal replacement medication (29%) followed by few side effects (25%) and low risk of adrenal crisis (22%). Similar responses were obtained from patients with primary and secondary AI.

### Health-related questions and hospitalizations

A majority of the patients (648 of 1026 [64%]) reported impacted quality of life (QoL) due to their illness, 87% (99 of 114) of patients with secondary AI and 60% (515 of 857) of the patients with primary AI. Approximately three quarters (73 of 99 [74%]) of the patients with secondary AI graded the impact on QoL as “quite a lot” or “very much”, Table 3. The proportion of patients who reported impaired QoL varied with dosing regimen (OD > BID > TID). However, the level of impairment did not differ between the dosing regimens. A lower proportion of patients treated with hydrocortisone reported impaired QoL compared with patients receiving cortisone acetate or prednisone/prednisolone but the level of impairment did not differ between the therapies (data not country-adjusted).

**Table 3:** Impact of adrenal insufficiency on quality of life (QoL) captured in an international survey

		**Primary AI**	**Secondary AI**	**All**
		**n (%)**	**n (%)**	**n (%)**
Impact on QoL	Yes	515 (60%)	99 (87%)	658 (64%)
	N	N = 857	N = 114	N = 1026
Degree of impact on QoL	A little	73 (14%)	10 (10%)	86 (13%)
	Intermediate	170 (33%)	16 (16%)	195 (30%)
	Quite a lot or very much	267 (52%)	73 (74%)	372 (57%)
	N	N = 510	N = 99	N = 653

**Figure 1:**
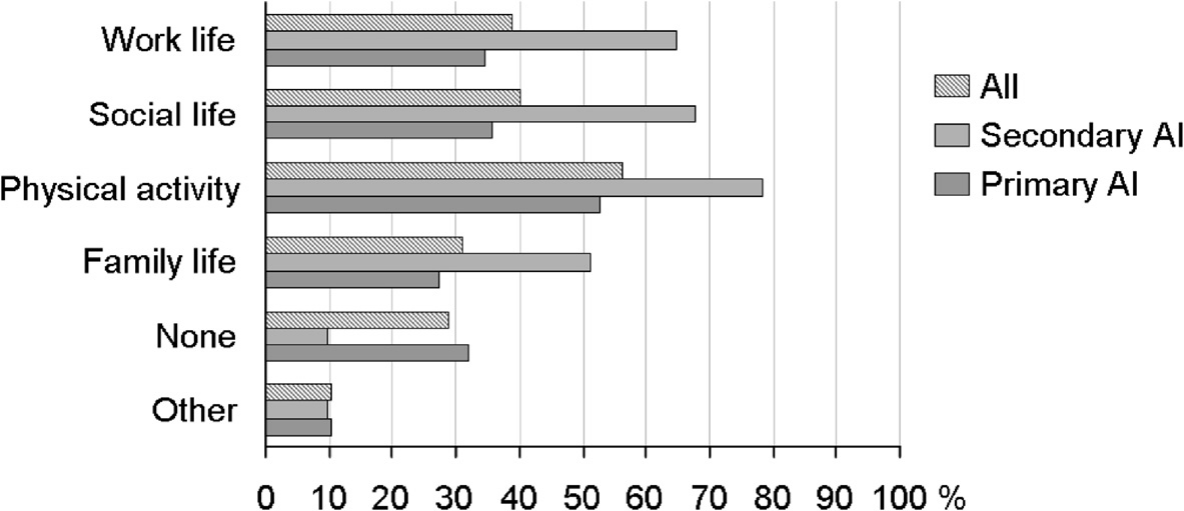
**Change in activities due to adrenal insufficiency.** Responses to the question “What activities do you need to alter due to your adrenal insufficiency”? in an international patient survey. A total of 1001 subjects responded to this question.

All patients were asked “What activities do you need to alter due to your AI?”, regardless of whether they had answered that their QoL was impaired or not. A large percentage of the participants reported that they had had to alter their physical activity (56%), social life (40%), work life (39%) or family life (31%) due to their illness (the participants were allowed to choose more than one alternative). A higher proportion of patients with secondary AI (90%) than with primary AI (68%) reported that they had had to alter work life, social life, physical activity or family life, Figure 1.

A majority of the participants reported fatigue in the morning (57%) and during the day (65%) to be a problem. Fatigue was more pronounced in patients with secondary AI than in patients with primary AI, Table 4. Of those reporting morning fatigue to be a problem, 75% also reported fatigue during the day to be a problem. Similarly, of those reporting fatigue during the day to be a problem, 85% also reported morning fatigue to be a problem.

**Table 4:** Fatigue in the morning and during the day reported by patients with adrenal insufficiency in an international patient survey

		**Primary AI**	**Secondary AI**	**All**
		n (%)	n (%)	n (%)
Fatigue experienced as a problem in the morning	Yes	438 (53%)	91 (81%)	571 (57%)
	N	N = 832	N = 113	N = 998
Fatigue experienced as a problem during the day	Yes	508 (61%)	99 (87%)	644 (65%)
	N	N = 830	N = 114	N = 995

In this survey, 61% of the respondents considered themselves fit to work while 17% did not. Additionally 5% of the respondents both considered themselves unfit to work and were on sick leave, 10% were retired and 7% were unemployed. Of those considering themselves fit to work, 72% considered themselves fit to work full-time, while 18% could work 75%, 9% could work 50% and 1% could work <50%. Overall, 40% of the participants had been absent from work in the last 3 months and, again, this was more common among patients suffering from secondary AI (50%) than for patients with primary AI (38%). Almost one third (28%) of those being away from work or school reported more than 3 weeks’ absence in the 3 months preceding their participation in the survey, Table 5. A higher percentage of patients treated with prednisone/prednisolone reported absenteeism compared with patients on hydrocortisone and they also reported more lengthy absenteeism (data not shown).

**Table 5:** Absenteeism from work or school due to adrenal insufficiency in the last 3 months reported in an international patient survey

		**Primary AI**	**Secondary AI**	**All**
		**n (%)**	**n (%)**	**n (%)**
Any abseenteism	Yes	299 (38%)	54 (50%)	379 (40%)
	N	N = 796	N = 108	N = 951
No. of days of abseenteism	1-5	174 (59%)	17 (33%)	198 (53%)
	6-10	37 (13%)	6 (12%)	46 (12%)
	11-15	16 (5%)	5 (10%)	23 (6%)
	>16	70 (24%)	24 (46%)	107 (29%)
	N	N = 297	N = 52	N = 374

A majority of the participants were worried about long-term side effects. The participants were most worried about osteoporosis (79%), followed by obesity (64%), fatigue (52%) and cardiovascular problems (46%) (more than one alternative could be chosen), Table 6. A higher proportion of those treated with prednisone/prednisolone were worried about long-term side effects than those treated with hydrocortisone (data not shown).

**Table 6:** Worries about long-term effects reported by patients with adrenal insufficiency in an international patient survey

		**Primary AI**	**Secondary AI**	**Respondents**
		**n (%)**	**n (%)**	**n (%)**
Any worry	Yes	612 (75%)	93 (82%)	740 (76%)
	N	N = 819	N = 113	N = 977
Pre-specified long-term effects	Osteoporosis	477 (79%)	76 (82%)	578 (79%)
	Obesity	374 (62%)	72 (77%)	464 (64%)
	Fatigue	301 (50%)	63 (68%)	381 (52%)
	Cardiovascular	258 (43%)	58 (62%)	334 (46%)
	Infectious disease	230 (38%)	52 (56%)	300 (41%)
	Other	117 (20%)	15 (16%)	138 (19%)
	N	N = 601	N = 93	N = 728

Overall, 32% of the participants reported that they increased their dose due to physical activity at least once per week (primary AI patients: 30%; secondary AI patients: 49%) and 66% increased their dose due to illness at least once per month (primary AI patients: 65%; secondary AI patients: 76%).

Overall, 38% of the participants responding to the question about hospitalizations (N = 977) answered that they had been hospitalized at least once during the last 12 months (37% of patients with primary AI and 43% of patients with secondary AI). One third of the patients (32%) had been hospitalized more than once during the last 12 months. The reported reason for hospitalization was adrenal crisis, vomiting or an acute infection for 17% of the patients who had been hospitalized.

## Discussion

The conduct of this survey was similar to previous cross-sectional surveys with participants recruited via patient organizations [5,11,12]. The current survey of 1245 respondents is to our knowledge the largest patient survey to date in patients with AI. Previous studies have shown that outcomes in patients with AI are compromised [12-14]. The responses from this survey provide more information on the impact of the disease and its treatment on patient-perceived outcomes, and support previous data showing a large impact of the disease and its treatment on the daily life of these patients.

Therapy traditions differ to some extent between countries but hydrocortisone was the most commonly used therapy in this survey (75%), irrespectively of country, which is also in line with previous studies from Europe [4,20]. The distribution of therapies, i.e. type of glucocorticoid, was similar between patients with primary and secondary AI. Three quarters of the patients were on a BID or TID regimen. One unexpected observation in this survey was the high percentage (58%) of patients on prednisone/prednisolone who were on a BID or TID regimen.

That multiple daily dosing is a problem from a compliance point of view is well known from other therapies [21]. The free-text answers to the open questions of this survey confirmed that multiple daily dosing is not optimal in AI and has an impact on patients’ social life and work life. More specifically, 4 of 10 patients in this survey found multiple daily dosing to be a problem. The impact on leading a normal life was attributed both to multiple dosing and to fluctuations in mental/physical energy over the day as multiple daily dosing with hydrocortisone/cortisone acetate causes peaks and low trough values in-between dosing occasions. When asked about the most important features of an optimal replacement medication, the patients ranked efficacy over 24 hours first and few side effects second. More patients on TID were satisfied with their treatment compared to those on BID or OD treatment. This might be attributable to the better cortisol coverage of TID during the active part of the day [22,23] over the convenience of fewer dosing times.

The results from this survey are in line with a recently published clinical study [24] which showed that a majority of the patients preferred the four-daily dosing regimen to twice daily when comparing equal doses of hydrocortisone given either twice daily or four times daily. The reasons reported were less fatigue, more alertness during the day, less headache and a feeling that the treatment effect was less varying during the day. The patients had complaints after the study that a four-dose regimen may be difficult to manage in the long run [24]. Another study has shown that a thrice-daily administration with weight-adjusted doses provides a better PK profile within the constraints of immediate-release hydrocortisone formulations [25]. 85% of the patients opted to remain on the TID regimen given in that study. These data suggest that patients experience benefits of having increased cortisol coverage during the active part of the day. One caveat of the above studies is that the total exposure of cortisol is higher when the same daily dose of hydrocortisone is administered divided into three or four daily doses than at BID administration. This is due to the fact that increasing the dose of hydrocortisone at one dose occasion does not result in a proportional increase in total exposure of cortisol due to the non-linear bioavailability of orally administered hydrocortisone [26]. Thus, there might be a short-term perceived benefit of the increased cortisol exposure whereas the long-term risk may increase.

It is difficult to mimic physiological cortisol profiles with immediate release hydrocortisone replacement therapies [22,25]. Therefore, attempts to better mimic the normal cortisol profiles have been made by developing new treatment regimens using the concept of chronotherapy, i.e. considering circadian rhythms in determining the timing and amount of the medication to optimize the desired effects and minimize the undesired ones [17,27]. A once-daily treatment with dual action, combining immediate release and extended release hydrocortisone has shown benefit over immediate release hydrocortisone with the same daily dosing administered thrice daily in patients with adrenal insufficiency [28].

Two thirds of the participants reported impacted QoL from their illness. In line with this being a patient survey (capturing the patient’s general perception of their subjective health at only one time point) and not a clinical trial, the questionnaire used in this survey did not include validated QoL questions from specific QoL questionnaires. Instead, the included questions were focused on the patients’ general perception of how and to what degree their disease and/or treatment affected their QoL. This survey showed that patients with secondary AI perceived their QoL as more impaired than patients with primary AI, which is in agreement with a previous study using validated QoL questionnaires showing that patients with AI have compromised QoL and that the impairment of QoL is worse in patients with secondary AI [14]. The current survey did not collect data on co-morbidities. Patients with secondary adrenal insufficiency may have other hormone deficiencies which could impact on QoL. However, data from previous studies are inconsistent and while some impact was observed in a Norwegian study of patients with Addison’s disease [13], another study [14] showed that the impairment in health-related subjective health status in AI patients is largely independent of concomitant diseases.

More than half of the AI patients needed to alter their physical activity and many needed to change their social life, work life or family life due to their illness. The level of impact on social life is in line with data from another international survey, conducted in 2003, in which one-third reported that their condition impacted on their ability to participate in social activities [29]. A Dutch patient survey reports similar impact on lifestyle [5]. Detailed questions on fatigue were included in the current survey as fatigue is the most commonly reported subjective health-related problem in AI [5,11-13,29,30]. This survey showed that 57% of the participants experienced fatigue in the morning as a problem and 65% reported fatigue during the day to be a problem. This response is in some contrast to the general perception that morning fatigue is a much larger clinical problem than fatigue during the day in patients with AI who have low or undetectable serum morning cortisol levels when using hydrocortisone BID or TID. Fatigue was more common in secondary AI than in primary AI and often necessitated changes in daily activities or changes in dose or the timing of dosing.

The current survey demonstrated a high absenteeism from work or school. More specifically, 4 of 10 patients reported absenteeism from work or school due to AI in the last 3 months and one third of those reported more than 3 weeks’ absence. Furthermore, 17% of the participants did not consider themselves to be fit to work. Among those who considered themselves fit to work, 28% worked part-time instead of full-time. These data are similar to those of a survey conducted across the UK, Canada, Australia and New Zealand (n = 850) [29], in which over 10% of the respondents reported that they were unable to work compared with 1% in the matched control group. Moreover, in the study by Hahner et al. [14], 18% of the AI patients (primary and secondary) did not work vs. 4% in the general population. In a Norwegian study, working disability amounted to 26% among patients with primary AI vs. 10% in the general population [13].

Patients with AI are educated to increase their glucocorticoid dose in stressful situations and in association with other illnesses. This survey showed that 60-75% of the patients increased their replacement dose due to illness every month. The high rate of hospitalizations in this survey (38% of the patients had been hospitalized at least once in the last year) is in line with the Dutch survey in which 3 of 10 patients had been hospitalized [5]. The reported reason for hospitalization was adrenal crisis, vomiting or an acute infection in 17% of the patients. As a comparison, the incidence rate of adrenal crises was 6.3 per 100 patient years in a study by Hahner et al. [31]. The reason for this discrepancy is most likely the differences in methods of collecting data and that the definition of adrenal crisis is not the same, e.g. only cases of hospitalization necessitating i.v. glucocorticoid administration were counted as adrenal crises in the study by Hahner.

Because of the relatively small disease population, age and gender were not included in the questionnaire in order to protect personal integrity and anonymity. Internet use and behaviours linked to it are hypothetical confounding factors in the interpretation of the survey results. Patients who seek information about their disease usually tend to be more active than other patients, which can also affect the results. Despite the fact that congenital adrenal hyperplasia (CAH) constitutes one form of primary AI, it would have been interesting to analyse data on this patient group separately as recent data published by Arlt et al. (2010) showed significantly impaired health status and adverse metabolic and skeletal health in adult CAH patients [32], however, data were not separately collected for patients with CAH in this survey.

## Conclusions

This international survey showed that the glucocorticoid replacement regimens in AI differ to some degree between countries. Three quarters of the AI patients participating in the survey received hydrocortisone administered two or three times daily. A large majority of the participants reported that their disease and the current treatment have an impact on QoL leading to alterations in physical activity, social life, work life and family life. Furthermore, 76% of the participants reported concerns of long-term side-effects of their treatment. These data demonstrate - from the AI patients' perspective – an obvious need for improvement in the management of AI including the regimen of glucocorticoid replacement therapy.

## Competing interests

This survey was funded by DuoCort Pharma. MF works for DuoCort Pharma AB. *GB, SS and GJ* have equity interests in DuoCort AB.

## Authors’ contributions

All authors contributed to the design of the survey and writing/review of the manuscript. All authors read and approved the final manuscript.

## Pre-publication history

The pre-publication history for this paper can be accessed here:

http://www.biomedcentral.com/1472-6823/12/8/prepub
